# Colonoscopic titanium clipping to address appendiceal stump leakage: a case report

**DOI:** 10.3389/fsurg.2023.1171875

**Published:** 2023-07-19

**Authors:** Jianjun Liu, Huayan Yuan, Xiulian Xu, Longkuan Yin, Wei Wang, Wenhai Fan, Xiangyu Bai, Pan Wang

**Affiliations:** ^1^ Department of Gastrointestinal Surgery, Institute of Hepatobiliology and Pancreaticoenterology of the Affiliated Hospital of North Sichuan Medical College, Nanchong, China; ^2^ North Sichuan Medical College, Nanchong, China; ^3^ Translational Medicine Research Center, North Sichuan Medical College, Nanchong, China

**Keywords:** appendiceal stump leakage, appendectomy, colonoscope, titanium clipping, case report

## Abstract

The incidence of appendiceal stump leakage (ASL) is extremely low and heterogeneous, which has been reported to be approximately 0.5%–1.0%. It is a catastrophic complication with high mortality rate despite its low morbidity. Once it occurs, it will put the doctor in a passive position because dealing with the leakage is much more cumbersome than appendectomy. We extensively reviewed the literature on ASL, focusing on the management and prognosis. Unsurprisingly, all of the physicians advocated extended resection, which apparently gave them sufficient confidence. However, partial cecum resection, cecostomy, or terminal ileectomy is extremely invasive and destructive. So, the patients had to experience great mental and physical trauma, longer hospital stays, higher rates of wound infection, more costs, and even a third surgery. Therefore, are there any better approaches for ASL? In this article, we report a case of ASL who successfully underwent endoscopic treatment. A 70-year-old male was admitted with gangrenous perforated appendicitis with a large iliopsoas abscess. Appendectomy, iliopsoas abscess debridement and sufficient drainage, appendicular stump repair and closure, and terminal ileostomy were performed. Three months later, the patient was readmitted and the stoma reversal was performed as scheduled. Seven days later, ASL was found when a liquid diet was applied routinely due to right lower quadrant pain and low fever. Finally, with the periappendiceal abscess completely drained, we clamped the appendiceal orifice with five titanium clips under an electronic colonoscope, which eventually sealed the leakage and avoided extended resection.

## Introduction

Appendicitis is the leading cause of acute abdomen, with an incidence of about 1‰ in the general population (1). One part of acute appendicitis would develop into periappendiceal abscesses within a few days if left untreated, and the other part may progress to severe acute abdomens such as perforated appendicitis with peritonitis, which makes subsequent treatment difficult (2, 3). Therefore, appendectomy has always been considered the standard treatment, and it could be performed by laparostomy or laparoscopic surgery if the patient has no contraindications to surgery. There are many surgical methods to deal with appendiceal stump and mesoappendix, including electrocautery, ligation, endostaplers, endoloops, endoclips, and so on (4–7). Even so, complications such as bleeding, infection, intra-abdominal abscess, appendiceal stump leakage (ASL), etc. may frequently occur. Among them, ASL is considered to be one of the most serious and difficult to deal with (8).

We reviewed extensive English language literature studies on appendectomy over the past 5 years via PubMed and Web of Science. Keywords were restricted to ASL, complications, and prognosis. Only a few literature reviews were found reporting ASL: Flores-Marin et al. (9) reported one case of ASL out of 158 patients with complicated appendicitis. Taguchi et al. (10) conducted a randomized controlled trial study and concluded that in patients with complicated appendicitis, the leakage rate was 9.5% in the laparoscopic surgery group compared to 12.8% in the open surgery group. All related literature studies indicate that ASL is devastating and current treatment are mainly abscess debridement, partial cecum resection, enterostomy (cecostomy or terminal ileostomy), and so on. This paper reports a case of complicated appendicitis who was admitted to hospital due to appendicitis with large iliopsoas abscess and was eventually cured by endoscopic titanium clipping of the appendiceal orifice.

## Procedure

**Figure 1:**
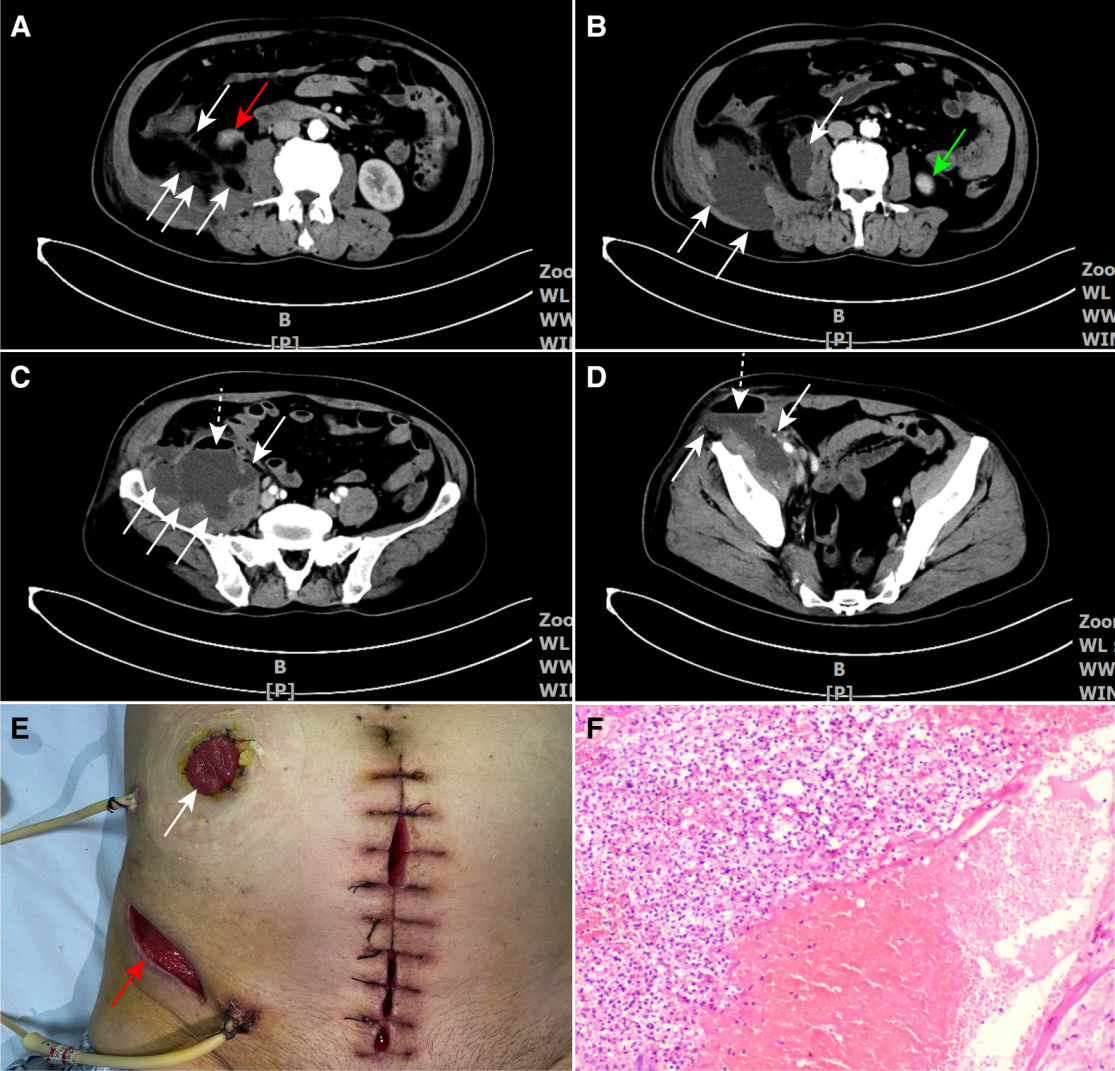
(**A**) The white arrows depict the gas-filled sections of the iliopsoas abscess. The red arrow points to the inferior pole of the right kidney. (**B–D**) CT scans reveal the extent of the iliopsoas abscess, with the white arrows outlining the abscess borders, the green arrow pointing to the inferior pole of the left kidney, and the white dotted arrows indicating the gas–fluid levels in the upper part of the abscess. (**E**) The postsurgery image displays the extra-abdominal area. The white arrow shows the stoma of the terminal ileum, and the red arrow highlights the incision made on the lateral side of the groin. (**F**) The postoperative pathology report confirms gangrenous appendicitis. CT, computed tomography.

A 70-year-old male complained of persistent and dull pain in the right lower quadrant for 20 days with intermittent chills and symptoms of high fever, diarrhea, palpitations, fatigue, and progressive right lower limb claudication. The patient was transferred to the General Surgery Department of the Chuanbei Hospital due to ineffective conservative treatment in the local community hospital on August 9, 2021. Routine blood test showed an increase in white blood cells (10.76 × 10^9^/L), and the percentage of neutrophils also rose to 92.30%. The abdominal enhanced computed tomography (CT) showed a large gas-containing irregular iliopsoas abscess between the right kidney, psoas major, and ascending colon, and the appendix with its root was almost completely decomposed (Figures 1A–C, Supplementary Figure S1A). The abscess originated from the inferior pole of the right kidney and descended to the deep right groin, and then extended into the inguinal lateral subcutaneous area to form a fluctuating mass (Figure 1D). On the second day after admission, the patient underwent open appendectomy (OA) with appendiceal root repair and closure, inguinal abscess incision and drainage, iliopsoas abscess debridement and drainage, and a terminal ileostomy (Figure 1E). Perforated gangrenous appendicitis was confirmed by the postoperative pathological examination (Figure 1F). The patient recovered with his right lower limb resumed activities and was discharged on the 10th day after surgery.

**Figure 2:**
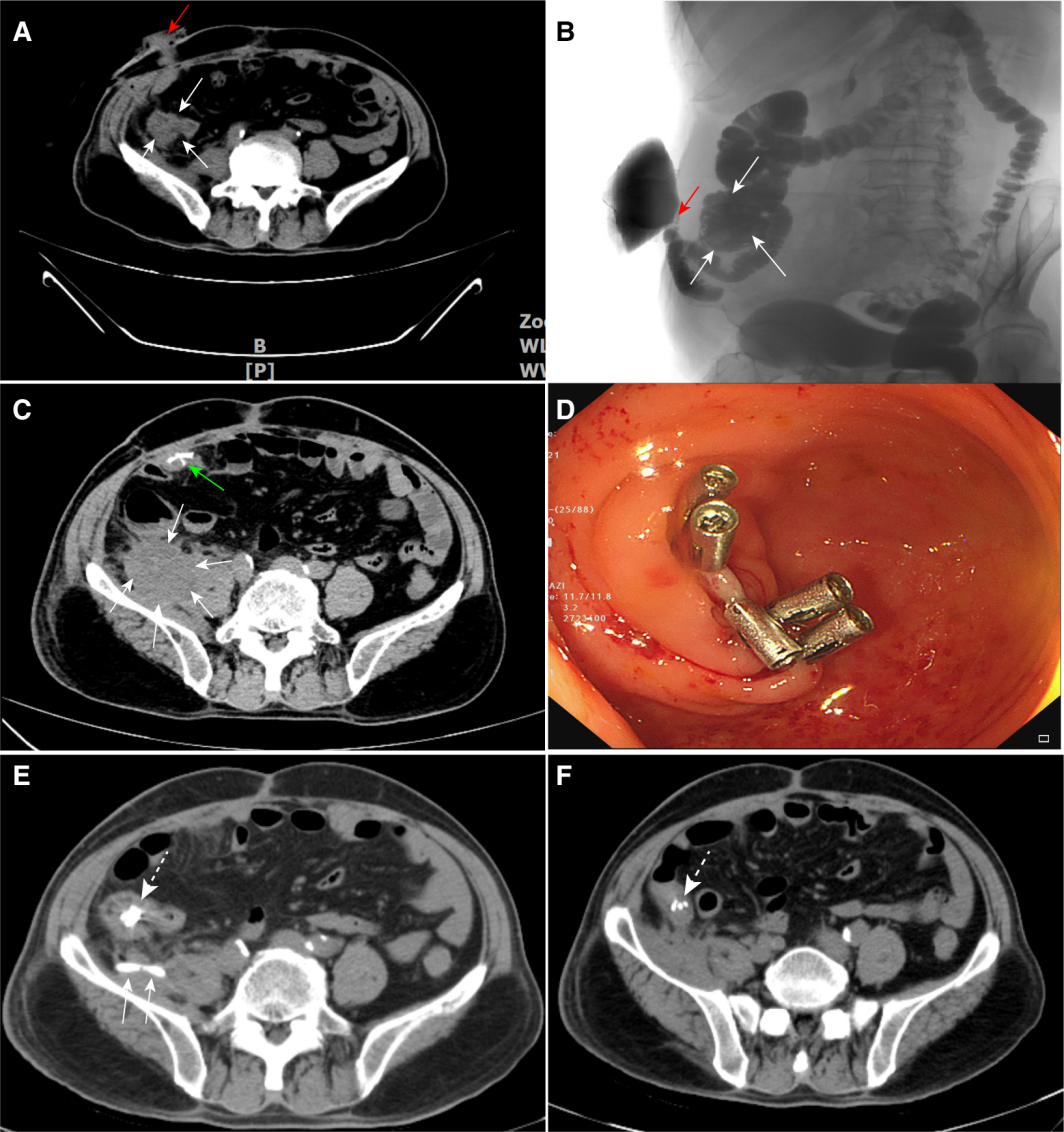
(**A**,**B**) Assessment of the baseline condition prior to reversal of ileal stoma: the white arrows indicate the cecum, and the red arrows represent the terminal ileal stoma. (**C**) The white arrows indicate the encapsulated abscess in the right iliac fossa, while the green arrow shows the titanium staples at the ileal anastomosis site. (**D**) Five titanium clips were clamped on the appendiceal orifice during colonoscopy. (**E**) On CT, the white dotted arrow indicates the titanium clips, and the white arrows display the J-catheter within the abdominal cavity. (**F**) Follow-up abdominal CT shows that the abscess has completely resolved. The white dotted arrow indicates the titanium clips. CT, computed tomography.

Three months later, the follow-up abdominal enhanced CT scan suggested that the iliopsoas abscess was completely absorbed (Figure 2A), and the colonic barium enema showed no contrast medium spillage from the appendiceal stump (Figure 2B, Supplementary Figure S1B). Therefore, the patient was readmitted and ileostomy reversal was performed subsequently. The patient started a full liquid diet on the third postoperative day, and the drainage tube was removed 2 days later. When the patient was about to be discharged on the seventh postoperative day, he complained of right lower quadrant pain and low fever. An emergency abdominal CT was carried out, which showed an encapsulated abscess (about 4.0 cm × 3.0 cm × 3.0 cm in size) around the appendiceal stump, indicating the presence of ASL (Figure 2C, Supplementary Figure S2A). A percutaneous insertion of an ultrasound-guided 7F J-catheter was performed to drain approximately 60 ml of fecal-odorous fluid from the abscess. Despite 5 days of conservative treatment, which involved fasting, gastrointestinal decompression, parental nutrition, and negative pressure drainage tube flushing, the leakage persisted. A follow-up abdominal CT scan confirmed the resolution of the abscess, but the ongoing daily discharge of the foul-smelling fluid indicated the inability of the leak to heal spontaneously. Hence, our dilemma was: to reoperate or pursue less invasive options?

After a comprehensive assessment, we decided to clamp the appendiceal orifice by titanium clipping under colonoscopy and open cecectomy as an alternative. In order to ensure effective blockage, five titanium clips (ROCC-D-26-195, Mirco-Tech, Nanjing) were closely arranged in a straight line and the orifice was sealed lip-shaped (Figures 2D,E, Supplementary Figure S2B). During the following 5 days of observation, no leakage was detected during the patient’s transition from a liquid diet to a solid diet and the J catheter was eventually removed. The results were unexpected and encouraging, and the patient was subsequently discharged with normal results during the 3 months of regular follow-up (Figure 2F).

## Discussion

There are broadly three treatment strategies for acute appendicitis according to different situations, namely, conservative treatment (antibiotics, pain killer, and long term follow-up), OA and laparoscopic appendectomy (LA). Approximately 30%–40% of uncomplicated cases who are in nonoperative care require appendectomy within 5 years (11). Decades ago, OA was the standard surgical procedure due to limited knowledge and medical conditions, especially in gangrenous or perforated appendicitis (12). From 2000 to 2015, LA has gradually replaced OA due to its advantages of shorter hospital stays, less pain, less trauma, lower infection rates, and so on (13), which is also recommended to pregnant patients in their first trimester (14). Nonetheless, if compromised appendicitis (gangrene or perforation) is found during LA, conversion to OA is recommended as appropriate (15).

Despite its low incidence and clinical rarity, ASL is considered one of the most serious complications of appendectomy. It cannot be avoided in spite of varies strategies to reduce the incidence of ASL through different reasonable stump closure methods. Once leakage appears, it will exacerbate the patient’s condition, followed by peritonitis, paralytic ileus, enterocutaneous fistula, and even septic shock, which may be life-threatening in severe cases (16, 17). Although there are several ways to minimize the harm of ASL, such as adequate drainage, delayed feeding, parenteral nutrition, etc., doctors are always frustrated when facing it. Thus, in order to minimize the incidence of such serious complications, appropriate caution is justified if gangrene or perforation is found when performing appendectomy.

In general, on-demand relaparotomy appears to be the most reasonable management option for ASL, including abscess debridement, partial cecum resection, and even enterostomy. After a certain period of time (no sooner than 60–90 days), the patient can undergo stoma reversal and fully return to the community and life (18). Although this approach is reasonable, the costs are enormous, and the patient suffers great loss and trauma, both psychologically and physically. Therefore, in addition to careful operation to minimize the occurrence of ASL, we should also keep in mind how to deal with it with minimal cost when it happens. In this case, we successfully blocked ASL by clamping the appendiceal orifice with five titanium clips in a straight line through colonoscopy, suggesting that this simple approach should be considered in certain cases. It depends on the size and integrity of the appendiceal orifice and may not be effective if it is severely damaged or deformed. Thus, in addition to puncture and catheter drainage of the periappendiceal abscess when ASL is present, we also recommend colonoscopy to assess the status of the appendiceal orifice and decide whether to clamp it, which would benefit to a large number of patients.

In conclusion, our report indicates that ultrasound-guided puncture and catheter drainage of the periappendiceal abscess and colonoscopic clamping of the appendiceal orifice with titanium clips are recommended for the treatment of ASL as long as the orifice is intact and of appropriate size. However, if the orifice is damaged or deformed, traditional surgical approaches should be considered such as partial cecum resection, enterostomy, and so on. This scheme has many incomparable advantages such as less trauma, low cost, better acceptance, etc., and has a good effect on the treatment of ASL.

## Data Availability

The original contributions presented in the study are included in the article/Supplementary Material, further inquiries can be directed to the corresponding author.
