# Erratum: Colonoscopic titanium clipping to address appendiceal stump leakage: a case report

**DOI:** 10.3389/fsurg.2023.1296548

**Published:** 2023-09-29

**Authors:** Frontiers Production Office

**Affiliations:** Frontiers Media SA, Lausanne, Switzerland

**Keywords:** appendiceal stump leakage, appendectomy, colonoscope, titanium clipping, case report

An erratum on Colonoscopic titanium clipping to address appendiceal stump leakage: a case report By Liu J, Yuan H, Xu X, Yin L, Wang W, Fan W, Bai X and Wang P (2023). Front. Surg. 10:1171875. doi: 10.3389/fsurg.2023.1171875

Due to a production error, there was an error regarding the title of the manuscript. Instead of “Colonoscopic titanium clipping to Maddress appendiceal stump leakage: a case report,” the title should be: “Colonoscopic titanium clipping to address appendiceal stump leakage: a case report.”

The publisher apologizes for this mistake. The original article has been updated.

