# Supplementary material for: Colonoscopic titanium clipping to address appendiceal stump leakage: a case report
Source: Front Surg. 2023 Jul 19;10:1171875. doi: 10.3389/fsurg.2023.1171875 (PMC10394468; doi:10.3389/fsurg.2023.1171875)

Supplementary Figure S1A Red circles show the extent and size of the ilio-psoas abscess alphabetically.

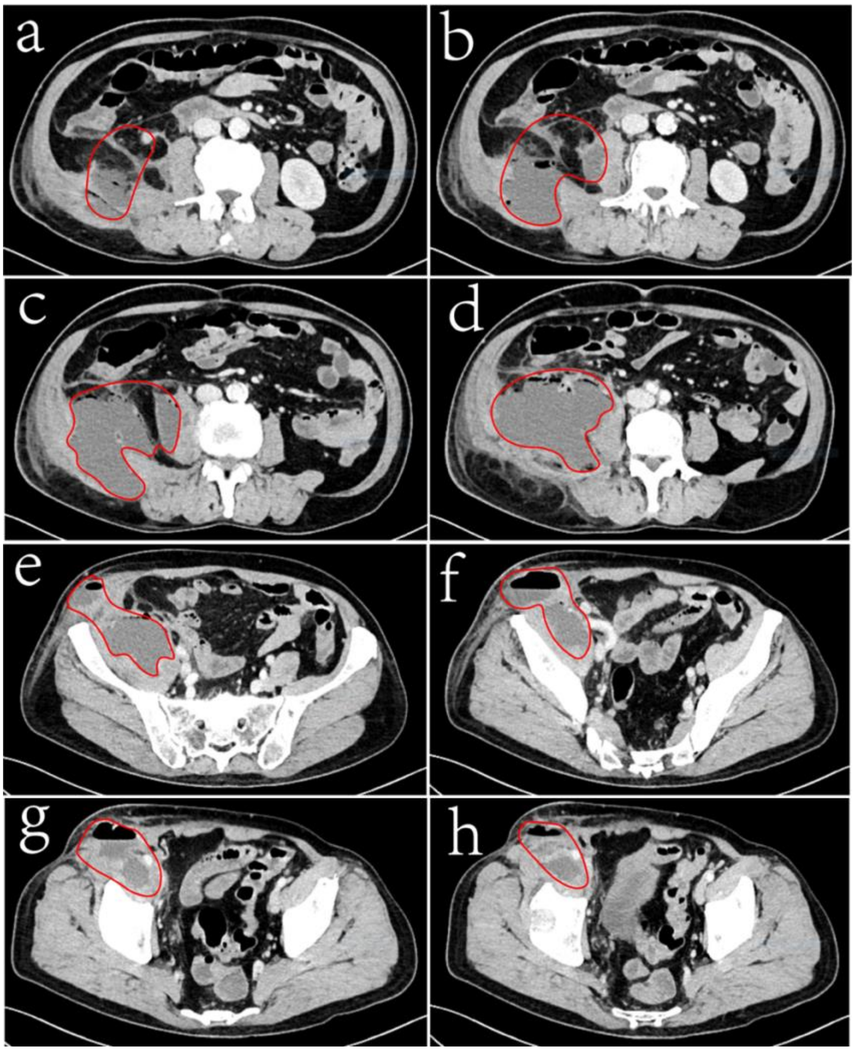

Supplementary Figure S1B Colonic barium enema shows no contrast medium spillage from the appendiceal stump in alphabetical order.

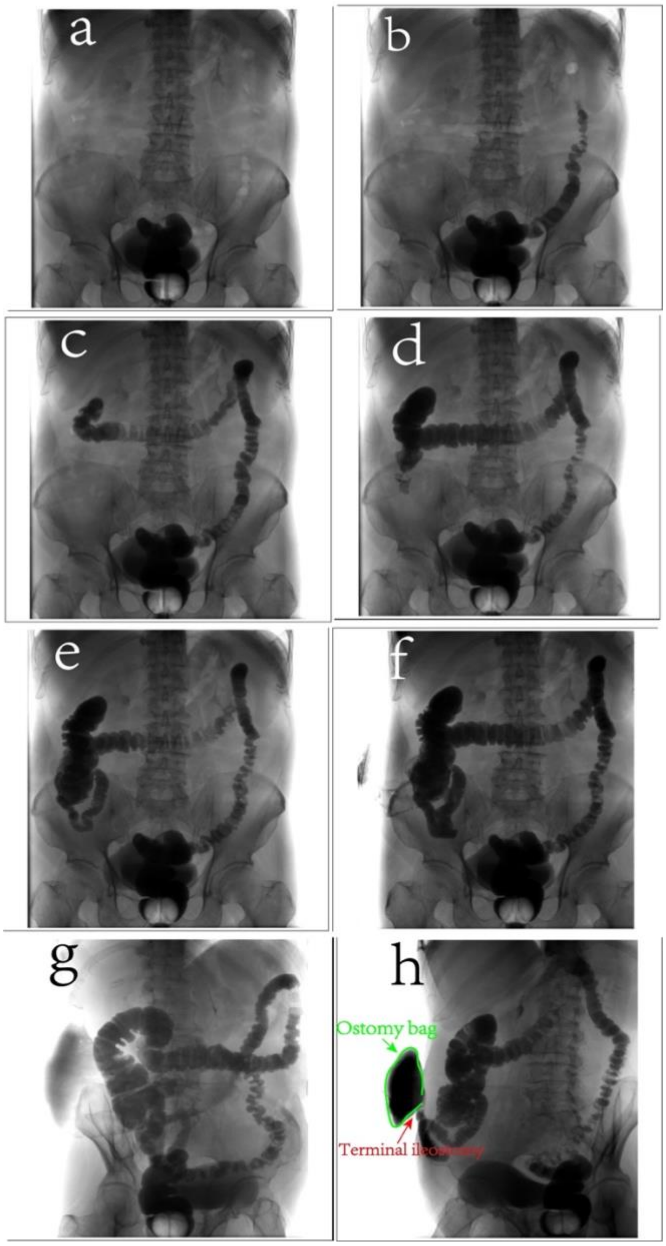

Supplement: Supplementary file 1 [file Datasheet1.pdf]
