# Supplementary material for: Colonoscopic titanium clipping to address appendiceal stump leakage: a case report
Source: Front Surg. 2023 Jul 19;10:1171875. doi: 10.3389/fsurg.2023.1171875 (PMC10394468; doi:10.3389/fsurg.2023.1171875)

Supplementary Figure S2A Red circles represent the encapsulated iliac fossa abscess around the ASL in alphabetical order. Green circles show the titanium staples on the ileal anastomosis.

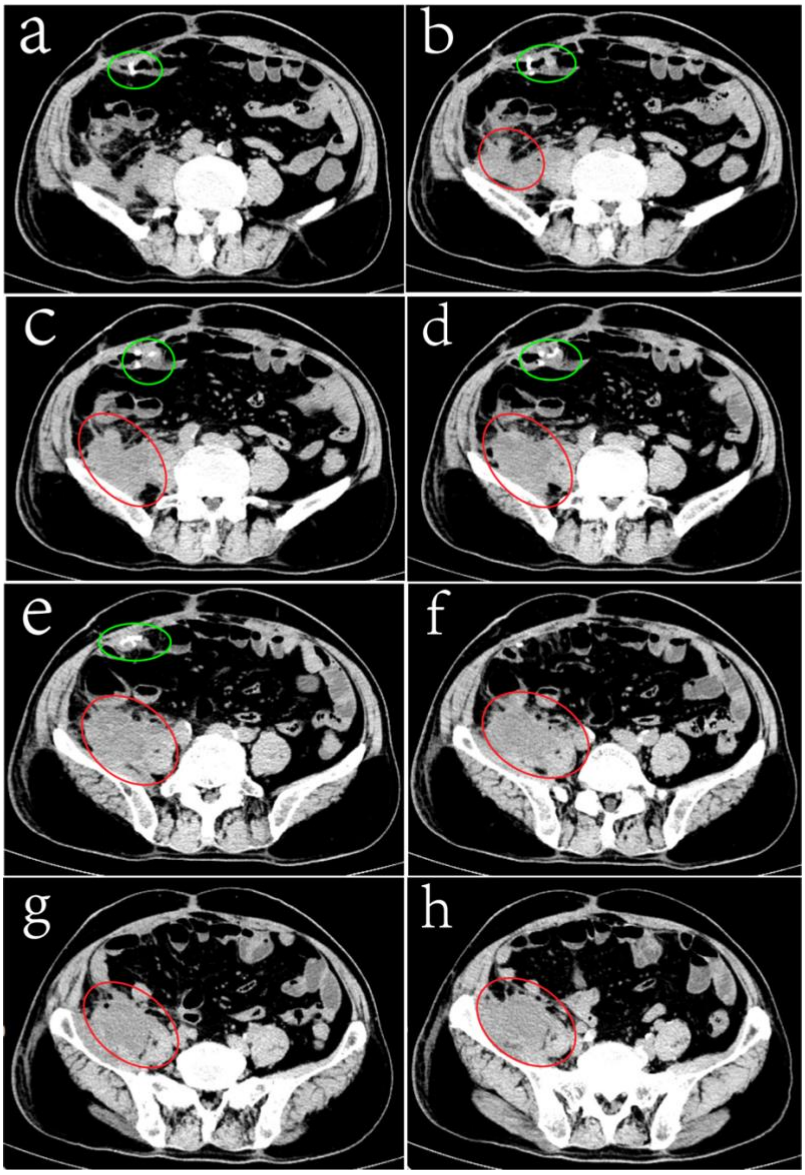

Supplementary Figure S2B Blue circles show the titanium clips on the appendiceal orifice. Greed circles show the ileal anastomosis. The red arrows show the path of the J catheter alphabetically.

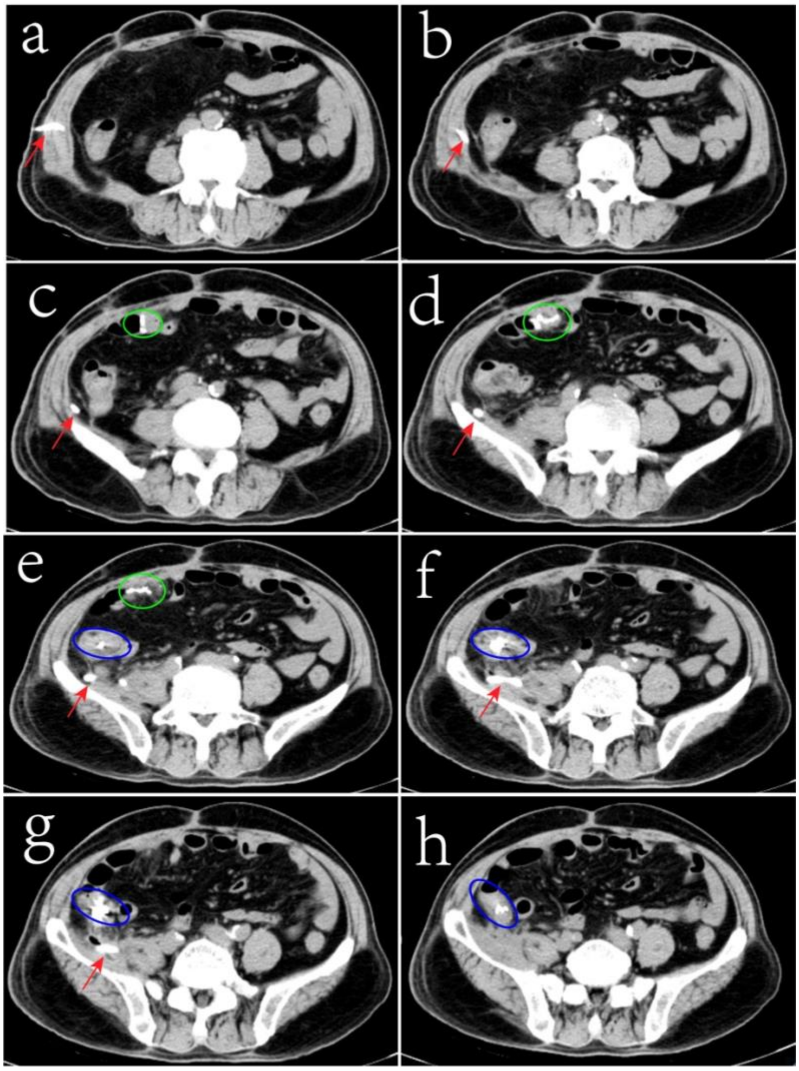

Supplement: Supplementary file 2 [file Datasheet2.pdf]
